# Contribution of ethnicity, area level deprivation and air pollution to paediatric intensive care unit admissions in the United Kingdom 2008–2021

**DOI:** 10.1016/j.eclinm.2024.102776

**Published:** 2024-08-16

**Authors:** Hannah K. Mitchell, Sarah E. Seaton, Christopher Leahy, Khurram Mustafa, Hannah Buckley, Peter Davis, Richard G. Feltbower, Padmanabhan Ramnarayan

**Affiliations:** a Section of Anaesthetics, Pain Medicine, and Intensive Care, Department of Surgery and Cancer, Faculty of Medicine, Imperial College London, London, England; b Institute of Child Health, University College London, London, UK; c Department of Population Health Sciences, University of Leicester, Leicester, UK; d PICANet, Leeds Institute for Data Analytics, School of Medicine, University of Leeds, Leeds, UK; e Paediatric Intensive Care Unit, Leeds Children's Hospital, Leeds, UK; f Paediatric Intensive Care Unit, Bristol Royal Hospital for Children, University Hospitals Bristol and Weston NHS Foundation Trust, Bristol, UK; g Children's Acute Transport Service, Great Ormond Street Hospital for Children NHS Foundation Trust, London, England

**Keywords:** Paediatric intensive care, Air pollution, Socioeconomic deprivation, Ethnicity, Health inequalities

## Abstract

**Background:**

There is emerging evidence on the impact of social and environmental determinants of health on paediatric intensive care unit (PICU) admissions and outcomes. We analysed UK paediatric intensive care data to explore disparities in the incidence of admission according to a child's ethnicity and the degree of deprivation and pollution in the child's residential area.

**Methods:**

Data were extracted on children <16 years admitted to UK PICUs between 1st January 2008 and 31st December 2021 from the Paediatric Intensive Care Audit Network (PICANet) database. Ethnicity was categorised as White, Asian, Black, Mixed or Other. Deprivation was quantified using the ‘children in low-income families’ measure and outdoor air pollution was characterised using mean annual PM2.5 level at local authority level, both divided into population-weighted quintiles. UK population estimates were used to calculate crude incidence of PICU admission. Incidence rate ratios were calculated using Poisson regression models.

**Findings:**

There were 245,099 admissions, of which 60.7% were unplanned. After adjusting for age and sex, Asian and Black children had higher relative incidence of unplanned PICU admission compared to White (IRR 1.29 [95% CI: 1.25–1.33] and 1.50 [95% CI: 1.44–1.56] respectively), but there was no evidence of increased incidence of planned admission. Children living in the most deprived quintile had 1.50 times the incidence of admission in the least deprived quintile (95% CI: 1.46–1.54). There were higher crude admission levels of children living in the most polluted quintile compared to the least (157.8 vs 113.6 admissions per 100,000 child years), but after adjustment for ethnicity, deprivation, age and sex there was no association between pollution and PICU admission (IRR 1.00 [95% CI: 1.00–1.00] per 1 μg/m^3^ increase).

**Interpretation:**

Ethnicity and deprivation impact the incidence of PICU admission. When restricting to unplanned respiratory admissions and ventilated patients only, increasing pollution level was associated with increased incidence of PICU admission. It is essential to act to reduce these observed disparities, further work is needed to understand mechanisms behind these findings and how they relate to outcomes.

**Funding:**

There was no direct funding for this project. HM was funded by an 10.13039/501100000272NIHR Academic Clinical Fellowship (ACF-2022-18-017).

**Research in context**

**Evidence before this study**

There is a growing body of evidence of the impact of social determinants of health on children's intensive care outcomes. Thorough searches of Medline and Embase databases were conducted from inception to 12th March 2024. Search terms included “P?ediatric∗”, “child∗”, “adolescen∗” AND “Critical care”, “intensive care” AND “race”, “racial”, “ethnic∗” OR “socio∗”, “deprivation”, “poverty”, “insur∗”, “income”, “Disparit∗”, “equity” OR “pollution” AND “incidence”. Key publications were supplemented with manual search of reference lists from papers in the literature to enrich the narrative review. There is evidence from the USA of increased incidence of Paediatric Intensive Care (PICU) admission in children residing in more deprived areas. Evidence from England has shown increased incidence of admission in children residing in more deprived areas and of South Asian ethnicity compared to White children and Children living in less deprived areas. There are little data investigating the association between air pollution and incidence of PICU admission.

**Added value of this study**

There have been changes in the levels of child poverty and pollution in the UK over the last decade, and changes in the proportions of different ethnic groups. There is an emerging understanding of the contribution of air pollution to childhood critical illness. Prior studies investigating disparities in incidence of admission to paediatric intensive care units in England and Wales use data more than 15 years old. This study provides an up-to-date analysis of admission trends and is the first paper to look at intensive care admissions across the whole of the UK and to explore the impact of air pollution on intensive care admissions.

**Implications of all the available evidence**

There is evidence of increased incidence of PICU admission in children of Asian and Black ethnicity and children residing in more deprived areas. When restricting to unplanned respiratory admissions and ventilated patients only, increasing pollution level was associated with increased incidence of PICU admission. Further work is required to develop community and hospital-based strategies to reduce disparities and understand how these disparities translate to outcomes after admission to paediatric intensive care.

## Introduction

Deprivation and ethnicity impact health in children and potentially their risk of admission to Paediatric Intensive Care Units (PICUs).^1^, ^2^, ^3^, ^4^ The majority of evidence investigating disparities in utilisation of paediatric intensive care comes from the United States of America.^1^^,^^2^ Studies from the USA have shown an increased incidence of admission to intensive care in children residing in more deprived areas.^5^, ^6^, ^7^ Evidence from the United Kingdom (UK) is more limited, with one study from England in 2006 finding that Asian and more deprived children had higher rates of PICU admission compared to other ethnic groups and children living in less deprived areas.^8^ A further UK study from 2005 showed that children admitted to PICU with traumatic brain injury were more likely to live in more deprived areas.^9^

The impact of air pollution on the incidence of admission to PICU is less well studied. Particulate matter (PM) is a key marker for air pollution; its concentration in the atmosphere reflects the presence of fine, inhalable particles from various pollution sources and is an indicator of the level of air quality. PM2.5 refers to fine particles less than 2.5 μm in diameter. Pollution has been associated with a range of adverse childhood outcomes globally.^10^ Increased exposure to PM has been associated with numerous adverse respiratory, neurologic and metabolic health outcomes in children born to mothers with a high level of antenatal PM2.5 exposure.^11^ Evidence from Europe showed children exposed to higher levels of PM2.5 had increased risk of developing persistent wheeze or asthma.^12^ In 2019, a third of UK local authorities had PM2.5 levels over the safe limit.^13^ There are currently no published studies investigating the association between air pollution and the incidence of PICU admission.

In this study we aimed to describe the incidence of PICU admission in the UK over a 14-year period to understand how social and environmental determinants of health influenced incidence of PICU admission. We aimed to understand how a child's ethnicity, area level deprivation and mean annual air pollution exposure are related to incidence of paediatric intensive care admission.

## Methods

### Study design and participants

This was a retrospective cohort study, using routinely collected Paediatric Intensive Care Audit Network (PICANet) data and UK population denominator information to describe the incidence of admission to PICU over a 14-year period.

All children, 0–15 years inclusive, admitted to UK NHS PICUs between 1st January 2008 and 31st December 2021 were included.

### Data sources

PICANet has been gathering information about all paediatric intensive care admissions from United Kingdom since 2008, including the child's characteristics, clinical condition and treatments received. Full details about the data collection can be found at: https://www.picanet.org.uk/data-collection/.

Office for National Statistics (ONS) Northern Ireland Statistics and Research Agency and National Records of Scotland provide mid-year population estimates broken down by age and sex at small area geography (Lower Layer Super Output Area (LSOA), Datazone and super output area (SOA)) level (population 500–3000 residents). Population denominators broken down by age, sex and ethnicity were released at the local authority (LA) level following the 2011 and 2021 census (population 2100–1,578,500 residents). 2011 census denominator populations were used for children admitted to PICU between 2008 and 2015 in England and Wales and Northern Ireland and 2021 denominator populations were used for children admitted to PICU between 2016 and 2021 in England and Wales and Northern Ireland. NRS do not plan to release 2022 census data broken down by age and ethnic group until summer 2024, so 2011 denominators were used for the entire period.

### Variables

The key exposure variables considered were the child's ethnicity, area level deprivation and mean annual pollution in the area that the child resided in at the time of PICU admission. Ethnicity of children admitted to PICU was extracted from PICANet and defined in line with UK government guidance^14^ as White (British, Irish and Other); Mixed (White and Black Caribbean etc); Asian (Indian, Pakistani, Bangladeshi and other); Black (Caribbean, African and other); Other (Chinese, Arab or other) and Unknown. Equivalent groups were used for the denominator population. Recognising that race does not have a basis in biology, so our study employs ethnicity not as an indicator of genetic or biological differences but as a reflection of the lived experiences of being an Asian or Black child in the UK.

Deprivation was measured using the ‘children in low-income measure’, a measure of families receiving governmental support,^15^ matched to LSOA/Datazone/SOA for crude and LA for adjusted analysis, assigned based on the child's postcode at the time of admission.

Fifths of deprivation were generated based on ranking the resident population (<16 years) of LSOA/Datazone/SOA or local authorities to ensure equal sized groups, with 1 being least deprived and 5 being most deprived.

Population-weighted mean annual PM2.5 by LA provided by Department for Environment, Food & Rural Affairs was used to define pollution exposure.^13^ This was available from 2010 to 2021, so all analyses investigating pollution are restricted to admissions after 2010. The UK has established a legal threshold for PM2.5 at 10 μg/m^3^, the previous limit was 20 μg/m^3^.^16^ Population weighted quintiles of pollution were created, with 1 being least polluted and 5 being most polluted, and crude incidence of PICU admission was presented in quintiles. Mean annual air pollution is included as a continuous variable, assigned based on the child's postcode at the time of admission.

Sex was categorised as male and female. Age at the time of admission was categorised as 0–4 years, 5–10 years, and 11–15 years in line with available denominator data.

### Statistics

We described the cohort of children and PICU admissions using descriptive statistics broken down by ethnic group. We reported summary statistics using means and standard deviations and medians and interquartile ranges. Categorical variables were summarised by frequency tables including counts and percentages. We graphically displayed the mean annual pollution and deprivation using choropleth maps.

To assess the impact of ethnicity, deprivation, and pollution on the rate of PICU admissions amongst the population, we reported crude PICU admission rates per 100,000 child years. Crude incidence was calculated for the key exposure variables by year of admission and presented graphically to assess changes over the study period. The data were checked to ensure there was a linear relationship between pollution and PICU admission and mean annual PM2.5 level was subsequently included as a continuous variable in later models.

We reported Incidence Rate Ratios (IRR) from Poisson multivariable regression models for all admissions combined, and then planned admissions and unplanned admissions separately. To account for children with multiple admissions, robust standard errors were adopted using a child-level identifier.^17^ Models were checked for overdispersion by calculating the ratio of the mean and the variance, if this was close to 1 it did not suggest overdispersion. We considered three exposure variables in relation to the main outcome variable (admission to PICU): air pollution, deprivation, and ethnicity. We used a causal inference approach and Directed Acyclic Graph (DAG) to identify the minimal sufficient adjustment set of confounders for each exposure (Supplemental Figure S1).^18^ For air pollution as the exposure, we adjusted for age, sex, ethnicity and deprivation. For deprivation we adjusted for age, sex, ethnicity; whilst for ethnicity we adjusted for age and sex.

A sensitivity analysis was done repeating the primary analyses restricting to children admitted to PICU who received invasive ventilation and with unplanned respiratory admissions only.

Pre-planned interaction tests were used to assess whether there was any evidence of effect modification of ethnicity by deprivation (model 1 included deprivation, ethnicity, age and sex, and model 2 included an interaction term between deprivation and ethnicity). If evidence of interaction was found, then results were presented stratified.

9.48% of children were missing information on the LSOA/Datazone or SOA they resided in. Children missing information on LSOA/Datazone or SOA were older than the cohort as a whole—LSOA was likely to have been withdrawn due to PICANet's anonymisation procedures so that geocoding would not be possible.^19^

Analyses were done using Stata version 18 (StataCorp, College Station, Texas, USA) and figures and maps were created in R. R Core Team (2023) (R: A Language and Environment for Statistical Computing. R Foundation for Statistical Computing, Vienna, Austria). DAGs and the minimal adjustment sets of confounders were produced and derived using DAGitty software version 3.1.^20^

### Ethical approval

As this study used de-identified, publicly available data, the NHS Health Research Authority Decision tool showed formal ethical approval was not required. As the dataset utilised in this study comprises routinely collected data for national clinical audit purposes individual patient consent was not required.

### Role of funding source

There was no direct funding for this project. HM was funded by an NIHR Academic Clinical Fellowship. The funder had no role in study design, data collection, data analyses, interpretation, or writing of report.

## Results

### Descriptive data

There were 245,099 admissions to PICU from 163,163 children between 2008 and 2021. Most admissions were unplanned (60.7%). Most children admitted were less than five years of age, with 14.6% of children admitted being <1 month of age (Table 1). Most children admitted to PICU were White (62.9%), 10.3% were Asian, 5.1% were Black and 2.8% were of other ethnicity. While 16% of all children were missing information on ethnicity, completeness of data improved over the study period; in 2021, 10.7% of children admitted were missing data on ethnicity. Children residing in areas with high child poverty and high pollution were overrepresented in the PICU admitted population with 23.7% and 21.2% of children residing in the most deprived and most polluted fifths respectively, compared to 13.6% and 15.6% residing in the least deprived and least polluted fifths. Children of Black and mixed ethnicity were more likely to reside in the most deprived quintile (44.7% and 42.6% respectively) compared to 19.3% of children of White ethnicity (Table 1). White children were least likely to live in the most polluted fifth (10.4%) compared to Asian and Black children (31.4 and 35.6% respectively).

**Table 1:** Characteristics of children admitted to paediatric intensive care by ethnic group (all admissions).

	Asian		Black		Mixed		Other		White		Missing		Total	
	(n = 25,236, 10.3%)		(n = 12,565, 5.1%)		(n = 7,224, 3%)		(n = 6,788, 2.8%)		(n = 154,053, 62.9%)		(n = 39,233, 16.0%)		(n = 254,099, 100%)	
	n	(%)	n	(%)	n	(%)	n	(%)	n	(%)	n	(%)	n	(%)
Sex														
Male	4063	56.2	14,463	57.3	7163	57.0	3862	56.9	87,152	56.6	22,225	56.7	138,928	56.7
Female	3161	43.8	10,771	42.7	5400	43.0	2924	43.1	66,885	43.4	17,002	43.3	106,143	43.3
Age group														
<1 Months	1058	14.7	3317	13.1	1191	9.5	806	11.9	22,778	14.8	6679	17.0	35,829	14.6
1–11 months	2379	32.9	7685	30.5	3581	28.5	2141	31.5	46,951	30.5	12,083	30.8	74,820	30.5
1–4 Years	2108	29.2	7043	27.9	3816	30.4	1973	29.1	39,941	25.9	10,024	25.6	64,905	26.5
5–10 Years	972	13.5	4272	16.9	2238	17.8	1115	16.4	22,564	14.7	4930	12.6	36,091	14.7
11–15 Years	707	9.8	2919	11.6	1739	13.8	753	11.1	21,819	14.2	5517	14.1	33,454	13.7
Area level deprivation quintile^a^														
1 (least deprived)	833	11.5	1458	5.8	578	4.6	644	9.5	23,823	15.5	6000	15.3	33,336	13.6
2	1015	14.1	1885	7.5	894	7.1	701	10.3	25,820	16.8	6371	16.2	36,686	15.0
3	1264	17.5	3443	13.6	1737	13.8	844	12.4	27,306	17.7	7285	18.6	41,879	17.1
4	1542	21.4	5437	21.5	3065	24.4	1075	15.8	29,237	19.0	8205	20.9	48,561	19.8
5 (most deprived)	2007	27.8	11,291	44.7	5348	42.6	2007	29.6	29,781	19.3	7678	19.6	58,112	23.7
Missing	563	7.8	1722	6.8	943	7.5	1517	22.4	18,086	11.7	3694	9.4	26,525	10.8
Average area level quintile^b^														
1 (least polluted)	613	9.5	1214	5.5	364	3.4	559	9.6	27,611	20.8	2758	8.1	33,119	15.6
2	820	12.8	3806	17.3	662	6.2	574	9.9	26,602	20.0	2875	8.5	35,339	16.7
3	1092	17.0	3284	14.9	1295	12.1	759	13.0	26,672	20.1	4672	13.8	37,774	17.8
4	1469	22.9	4743	21.5	1919	17.9	929	16.0	23,358	17.6	7147	21.1	39,565	18.7
5 (most polluted)	2020	31.4	7854	35.6	5865	54.6	1786	30.7	13,809	10.4	13,576	40.0	44,910	21.2
Missing	414	6.4	1143	5.2	640	6.0	1218	20.9	14,666	11.1	2910	8.6	20,991	9.9
Primary diagnosis group														
Cardiovascular	2169	30.0	7431	29.5	3059	24.4	2062	30.4	43,973	28.5	9538	24.3	68,232	27.8
Gastrointestinal	481	6.7	1540	6.1	683	5.4	414	6.1	9426	6.1	2781	7.1	15,325	6.3
Infection	327	4.5	1236	4.9	556	4.4	330	4.9	8360	5.4	2286	5.8	13,095	5.3
Neurological	738	10.2	2766	11.0	1386	11.0	762	11.2	16,931	11.0	4663	11.9	27,246	11.1
Other^c^	1293	17.9	4763	18.9	2536	20.2	1327	19.6	33,107	21.5	9012	23.0	52,038	21.2
Respiratory	2216	30.7	7500	29.7	4345	34.6	1893	27.9	42,256	27.4	10,953	27.9	69,163	28.2
Admission type^d^														
Planned	2673	37.0	8872	35.22	4118	32.8	2790	41.1	63,477	41.2	14,285	36.4	96,215	39.3
Unplanned	4550	63.0	16,335	64.7	8427	67.1	3994	58.8	90,450	58.7	24,890	63.4	148,646	60.7

a By small area geography (Lower layer Super Output Area, datazone, Super Output area).

b 2010–2021 only.

c Other includes blood/lymphatic, Body wall and cavities, Endocrine/metabolic, Multisystem, musculoskeletal, oncology, other and trauma.

d 238 records missing information whether admission planned or unplanned.

**Fig. 1:**
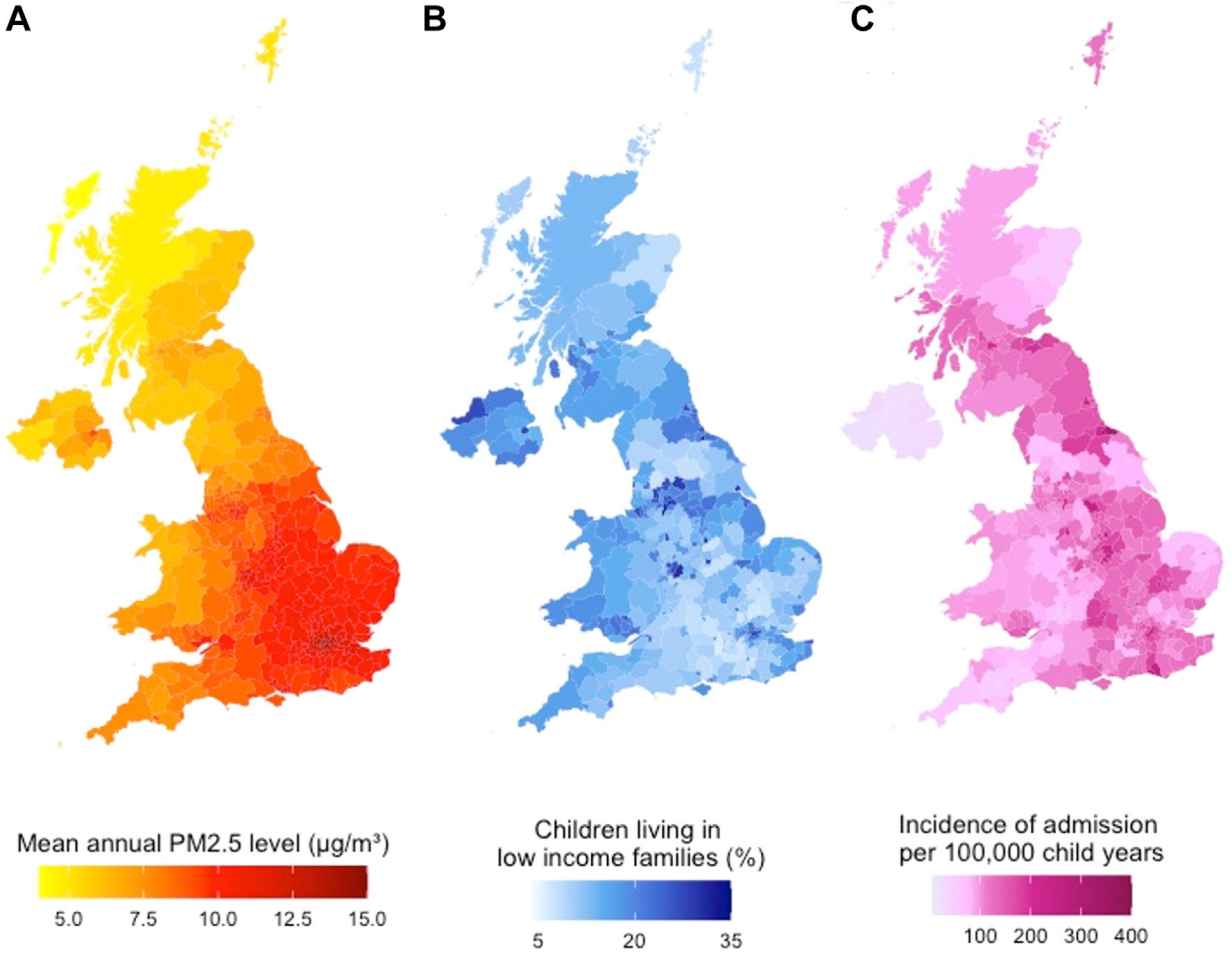
**A) mean Annual PM2.5 Exposure, B) mean percentage of children living in poverty and C) crude incidence of admission per 100,000 children by local authority (n = 374)**.

The most common primary diagnosis category for admission was respiratory (28.2%) followed by respiratory (27.8%) (Table 1). Outside London, the Southeast had a relatively smaller percentage of low-income families, but higher levels of air pollution (Fig. 1). Higher levels of deprivation were seen in Northern Ireland and the Northwest of England.

### Individual ethnicity

**Fig. 2:**
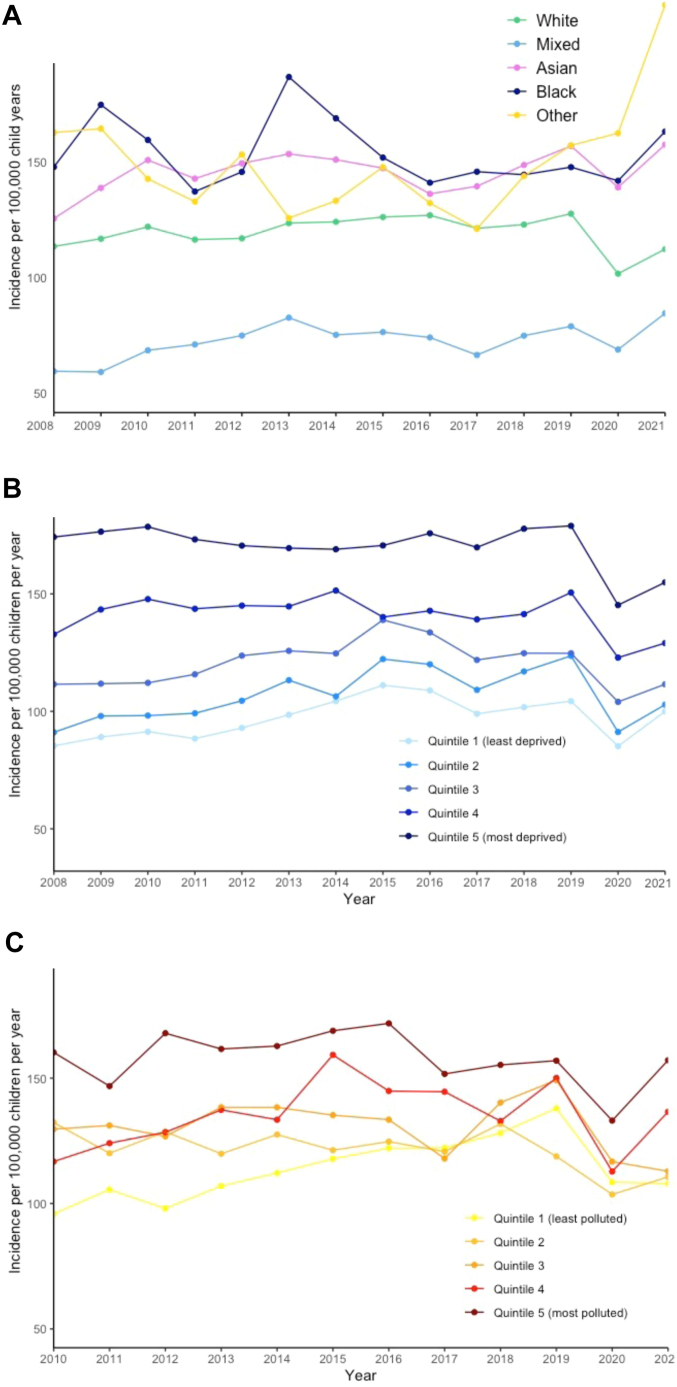
**Crude incidence of any admission to paediatric intensive care over time by A) ethnicity, B) quintile of deprivation (at lower layer superoutput area level) (children living in low-income families measure) and C) mean annual PM2.5 level (at local authority level) (NB y axis does not start at 0)**.

A higher crude incidence of PICU admission was observed in Asian children (145.7 per 100,000 child years [95% CI: 143.9–147.5]), Black children (154 per 100,000 child years [95% CI: 151.3–156.7]) and children of other ethnicity (150.5 per 100,000 child years [95% CI: 146.9–154.1] compared to White children ((119.8 per 100,000 child years [95% CI: 119.2–120.4]) (Table 2). There was increased crude incidence of admission to PICU in children of Asian, Black and Other children throughout the study period relative to White children (Fig. 2). Asian Pakistani children had the highest incidence of admission (189.2 per 100,000 child years [95% CI: 185.9–192.6]), followed by Black Caribbean, Other Asian and Black African children with 166.1, 161.3 and 155.1 unplanned admissions per 100,000 child years respectively (Supplemental Table S1).

**Table 2:** Crude incidence of admission to paediatric intensive care (per 100,000 child years) by ethnicity, deprivation and pollution by admission category.

	All admissions		Planned admissions		Unplanned	
	(n = 245,099)		(n = 96,215)		(n = 148,646)	
	Incidence	95% CI	Incidence	95% CI	Incidence	95% CI
Ethnicity						
White	119.5	(118.9–120.1)	49.3	(48.9–49.6)	70.2	(69.7–70.6)
Asian	145.7	(143.9–147.5)	51.2	(50.1–52.3)	94.3	(92.8–95.7)
Black	154.0	(151.3–156.7)	50.5	(48.9–52.0)	103.3	(101.1–105.5)
Mixed	72.6	(70.9–74.3)	26.9	(25.9–27.9)	45.7	(44.4–47.1)
Other	150.5	(146.9–154.1)	61.9	(59.6–64.2)	88.5	(85.8–91.3)
Area level deprivation fifth^a^						
1 (least deprived)	97.4	(96.3–98.5)	40.7	(40–41.5)	56.5	(55.7–57.4)
2	107.1	(106–108.3)	44.3	(43.5–45)	62.8	(61.9–63.6)
3	120.5	(119.3–121.7)	48.8	(48–49.5)	71.6	(70.7–72.6)
4	141.0	(139.8–142.3)	54.0	(53.3–54.8)	86.9	(85.9–87.9)
5 (most deprived)	170.2	(168.9–171.5)	60.1	(59.3–60.9)	109.9	(108.9–111)
Air pollution fifth^b^						
1 (low)	113.6	(112.4–114.9)	48.7	(47.9–49.5)	64.9	(64–65.8)
2	121.6	(120.4–122.9)	47.7	(46.9–48.5)	73.9	(72.9–74.9)
3	130.8	(129.5–132.1)	50.6	(49.8–51.4)	80.1	(79–81.1)
4	135.1	(133.7–136.4)	51.4	(50.6–52.2)	83.5	(82.4–84.5)
5 (high)	157.8	(156.4–159.3)	56.0	(55.2–56.9)	101.6	(100.4–102.8)

a By small area geography (Lower layer Super Output Area, datazone, Super Output area).

b Restricted to 2010–2021.

After adjusting for age and sex, Asian and Black children had higher relative incidence of PICU admission compared to White (IRR 1.17 [95% CI: 1.14–1.20] and 1.31 [95% CI: 1.27–1.36] respectively) (Table 3). When restricting to planned admissions only, there was no evidence of increased relative incidence of PICU admission in Asian or Black children (IRR 1.00 [95% CI: 0.97–1.03] and 1.04 [95% CI: 0.99–1.09]). When restricting to unplanned admissions only there was evidence of increased incidence of admission in children of Asian, Black and other ethnicity (IRR 1.29 [95% CI: 1.25–1.33], IRR 1.50 [95% CI: 1.44–1.56] and IRR 1.24 [95% CI: 1.18–1.30]). Across all admission types, children of mixed ethnicity had lower rates of PICU admission (Tables 2 and 3).

**Table 3:** Incidence rate ratio (IRR) of admission to paediatric intensive care for ethnicity, deprivation and pollution for all, planned and unplanned admissions.

	All			Planned			Unplanned		
	IRR	95% CI	P-value	IRR	95% CI	P-value	IRR	95% CI	P-value
Ethnicity^a^									
White	1 ref			1 ref			1 ref		
Asian	1.17	(1.14–1.20)	<0.001	1.00	(0.97–1.03)	0.866	1.29	(1.25–1.33)	<0.001
Black	1.31	(1.27–1.36)	<0.001	1.04	(0.99–1.09)	0.093	1.50	(1.44–1.56)	<0.001
Mixed	0.54	(0.52–0.56)	<0.001	0.49	(0.46–0.51)	<0.001	0.58	(0.55–0.60)	<0.001
Other	1.24	(1.19–1.29)	<0.001	1.23	(1.17–1.30)	<0.001	1.24	(1.18–1.30)	<0.001
Area level deprivation fifth^b^									
1 (least deprived)	1 ref			1 ref			1 ref		
2	1.05	(1.02–1.07)	<0.001	1.07	(1.03–1.11)	<0.001	1.04	(1.01–1.07)	0.016
3	1.13	(1.10–1.16)	<0.001	1.16	(1.12–1.21)	<0.001	1.11	(1.08–1.14)	<0.001
4	1.28	(1.25–1.32)	<0.001	1.28	(1.24–1.32)	<0.001	1.29	(1.25–1.33)	<0.001
5 (most deprived)	1.50	(1.46–1.54)	<0.001	1.32	(1.27–1.37)	<0.001	1.62	(1.57–1.66)	<0.001
Air pollution (per 1 mg/m^3^ increase)^c^	1.00	(1.00–1.00)	0.579	1.00	(1.00–1.01)	0.048	1.00	(0.99–1.00)	0.015

a Adjusted for sex and age (2008–2021).

b Adjusted for sex, age and ethnicity (2008–2021).

c Adjusted for sex, age, ethnicity and deprivation (2010–2021).

### Area level deprivation

Crude incidence of admission in the least deprived quintile was 97.4 per 100,000 child years, compared to 170.2 per 100,000 child years in the most deprived quintile. Throughout the study period there was increased incidence of PICU admission in children residing in the most deprived quintile relative to those in the least deprived quintile (Fig. 2).

After adjusting for age, sex, and ethnicity, there was 1.50 times the incidence of admission in children residing in the most deprived quintile compared to the least (95% CI: 1.46–1.55) across all PICU admissions (Table 3). There was increased relative incidence of admission in children living in more deprived areas in both planned and unplanned admissions, however the relative incidence rate was highest in unplanned admissions (IRR 1.62 [95% CI: 1.57–1.66]).

### Mean annual pollution exposure

Between 2010 and 2021 there was evidence of higher crude incidence of admission in children living in more polluted local authorities, with 113.6 (95% CI: 112.4–114.9) admissions per 100,000 child years for children living in the least polluted quintile compared to 157.8 (95% CI: 156.4–159.3) in children in the most polluted quintile (Table 2).

After adjusting for age, sex, ethnicity, and deprivation, there was no evidence of any difference in the risk of PICU admission according to levels of mean annual pollution exposure (Table 3).

### Stratified analysis

**Fig. 3:**
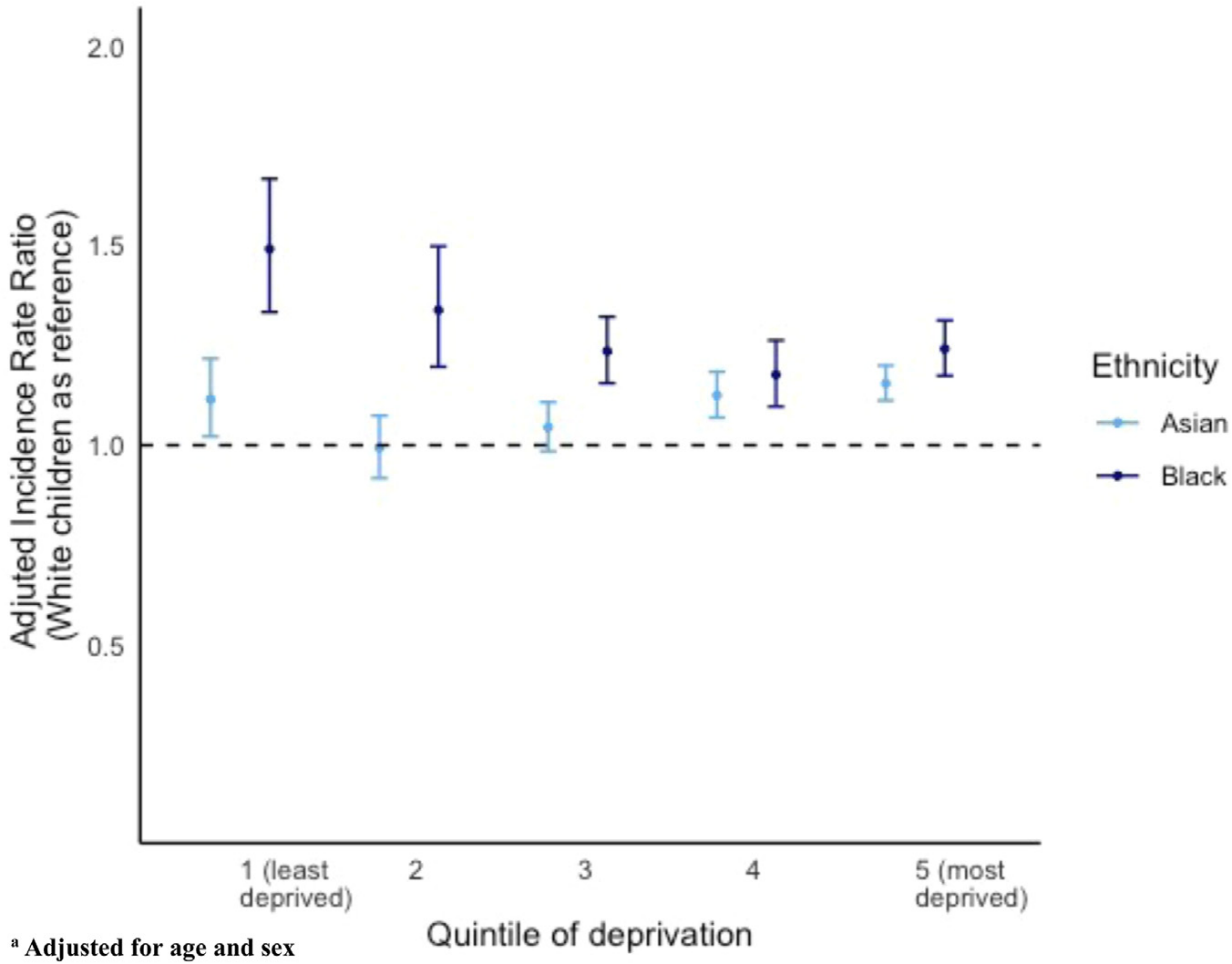
**Adjusteda incidence rate ratio of admission (error bars = 95% confidence intervals) to paediatric intensive care in Asian and Black children (White as reference)stratified by quintile of deprivation**.

There was evidence of modification of the effect of deprivation on the risk of PICU admission dependent on ethnicity (interaction test p < 0.001). Black children had significantly higher relative incidence of admission to PICU across all quintiles of deprivation (Fig. 3 and Supplemental Table S3). The relative rate of admission in Black children was highest in the least deprived quintile (IRR 1.49 (95% CI: 1.33–1.67).

### Sensitivity analysis

When restricting to patients who were either unplanned respiratory admissions or received invasive ventilation on PICU, similar results were seen for the relative incidence rates for ethnicity and deprivation, however there was evidence of an increase in admission incidence with every 1 μg/m^3^ increase in mean annual PM2.5 after adjusting for age, sex, deprivation and ethnicity (IRR 1.01 [95% CI: 1.00–1.01] and 1.02 [95% CI: 1.02–1.02] respectively (Supplemental Table S2).

## Discussion

In this comprehensive analysis, we explore the influence of ethnicity, socioeconomic deprivation, and air pollution on the incidence of PICU admissions across the United Kingdom from 2008 to 2021. There is a clear relationship between ethnicity and deprivation on children's intensive care admission. We found evidence of increased incidence of PICU admission in children of Asian and Black ethnicity. This was driven by increased incidence of unplanned admission in Asian and Black children, with no association between ethnicity and incidence of planned admission. Minority ethnicity conferred an increased incidence of unplanned admission to PICU independent of deprivation. Increasing deprivation conferred a significantly increased incidence of both planned and unplanned admission to PICU which persisted after adjusting for ethnicity and pollution. After adjusting for ethnicity and deprivation the pollution level of the area a child lived was no longer associated with increased incidence of PICU admission, however when restricting to unplanned respiratory admissions and ventilated patients only, increasing pollution level was associated with increased incidence of PICU admission after accounting for ethnicity and deprivation.

Our findings regarding the association of deprivation with admission to PICU is consistent with prior data from the UK and the US.^5^, ^6^, ^7^, ^8^ 2008 marked the Global Financial Crash with subsequent austerity policies that widely affected public services, including the NHS. Child poverty has increased in the UK since 2010, and in 2020 was at pre-2010 levels.^21^, ^22^, ^23^ Currently 4.2 million children are being raised in poverty in the UK, which equates to one in three children, with the recent increase in child poverty primarily driven by a rise in poverty among working families.^22^^,^^24^ Child poverty is associated with a range of adverse health outcomes—including low birth weight and asthma,^25^, ^26^, ^27^ which could predispose children living in poverty to become more critically unwell. The recent release of the National Child Mortality Database showed higher levels of mortality in children residing in more deprived areas.^26^ Our study shows children living in areas with high levels of child poverty have substantially increased incidence of critical illness across both planned and unplanned admissions. This alarming finding should be significant concern to policy makers and clinicians, prompting action to address this important issue.

Research from England and Wales (2004–2007) demonstrated higher incidence of admission in children of South Asian ethnicity.^8^ Our study includes the whole of the UK and analyses more recent data over a longer period accounting for changes in demographic profile of UK in recent decade and demonstrates increased relative incidence of admission in Black and Asian children compared to White children. There are higher rates of poverty in some ethnic groups—specifically Asian Pakistani and Asian Bangladeshi children, who also had higher rates of PICU admission. When investigating incidence of admission in specific ethnic groups, Asian Pakistani children had substantially higher incidence of PICU admission, followed by Black Caribbean, and Black African children.

Our study shows persistent evidence of differential unplanned admission by ethnicity after adjusting for deprivation and pollution. Racial and ethnic disparities in health outcomes have been demonstrated in children residing in the UK, including higher infant mortality in certain ethnic groups, ethnic disparities in COVID outcomes and following congenital heart surgery.^28^, ^29^, ^30^ The recent release of the National Child Mortality Database showed Black and Asian children had double the estimated child death rate compared to White.^26^ These findings reflect the international literature with evidence of worse acute care outcomes in children of minority ethnicity, refugee status and from indigenous populations.^1^ It is essential to understand why children Black and Asian ethnicity are overrepresented in intensive care, and how this translates to patient outcomes. The reasons for increased admission, including any barriers to health care or differential provision of health care need to be understood, so interventions can be developed to ensure more equal outcomes.

There is a lack of evidence on the association between pollution and PICU utilisation; however, one study from China demonstrated an association between small particle pollution exposure and ventilator associated pneumonia in children in PICU who had undergone cardiac surgery.^31^ In our univariable analysis we found increased incidence of PICU admission in children living in more polluted areas, however the effect of pollution was no longer significant after adjusting for deprivation. However, in a sensitivity analysis when we restricted only to unplanned respiratory admission and patients requiring invasive ventilation in PICU, we observed a 1–2% increase in relative incidence of PICU admission for every 1 μg/m^3^ increase in mean annual PM2.5 exposure, this would equate to a 20% increase in relative incidence of PICU admission between the most and least polluted local authorities in the UK. Specific diseases like asthma and wheeze should be explored further in future studies. Further work looking in a more granular fashion at the impact in time and space of air pollution on PICU admission is warranted.

Our study has some limitations over 10% of admitted patients were missing data on individual ethnicity, and around 10% of the cohort was missing information on small geographic area that they resided in. Ethnicity is recorded in PICANet in a variety of ways, either from self identification by patient or carers, medical records, and assignment by medical staff, whereas ethnicity in our UK population estimates is by self-report in the census. There are potential issues of misclassification, where families identify as mixed or other ethnicity, but they have been classified differently in health records. We suspect differential recording of mixed ethnic groups by ONS and PICANet due to low estimates of admissions in children of mixed ethnicity, so we were not able to reliably provide incidence estimates for this group of children. Although we were able to adjust for some confounders, there is likely to be residual confounding. Deprivation and pollution were considered at the area level and may not accurately capture a child's individual level of deprivation and pollution exposure. We use area level child poverty as a proxy for individual socioeconomic position, however there may be non-differential misclassification of socioeconomic position with children personally experiencing child poverty but living in a more affluent area, we would expect this to bias our effect estimate towards the null so effects could be more marked than we report. The effects of living in a high poverty area might have effects on PICU admission, separate from individual socioeconomic position.

The measure that we used to characterise pollution was mean annual PM2.5 level by LA, which is not a granular measure and does not allow us to account for changes in pollution over space and time. Our study does not accurately capture the effect of short-term changes in pollution, or account for the effects of living close to particularly polluted areas. Although our study did not find an association between PICU admission and pollution when looking at all admissions, the measure we use may mask the association and a true association may exist. Indoor air quality is known to have a deleterious effect on child health, and this is also not captured by our study.^32^ Future work should investigate the relationship between PICU admission and pollution modelling the effect of changes in pollution over short periods of time and space to understand the effect on PICU admission, and to understand association with reasons for admission and outcomes.

The limitations of our study are offset by some important strengths. We use a large national dataset with comprehensive coverage including 14 years of data with high ascertainment and data quality. All nations of the UK contribute to PICANet making our findings representative. We were able to adjust separately for each of our main exposures of ethnicity, deprivation and air pollution to try to disentangle the impact of social and environmental determinants of health. We used children living in low-income families measure to describe deprivation—which allowed comparison between England, Wales, Northern Ireland and Scotland. This measure is also directly relevant to children.

This work identifies a relationship between ethnicity and deprivation and incidence of children's intensive care admission. Further work is needed to understand mechanisms behind this and how they relate to outcomes. Understanding these mechanisms is crucial for developing targeted interventions aimed at reducing disparities and improving health outcomes for all children.

## Contributors

HM, SS and PR devised and designed study, planned the analysis and critically revised the paper. HM and SS verified the underlying data. HM drafted the paper. HB, CL, KM, PD and RF critically revised the paper. All authors read and approved the final version of the manuscript.

## Data sharing statement

The data used in this study are available from the Paediatric Intensive Care Audit Network (PICANet).

## Declaration of interests

Peter Davis has a paid role as Clinical Reference Group Chair for Paediatric Critical Care until 07/2022 and an unpaid role as Clinical Reference Group Member for Paediatric Critical Care from 08/2022. Sarah Seaton is funded by the Healthcare Quality Improvement Partnership (and equivalent funders from Scotland, Wales, Northern Ireland and Republic of Ireland) in her role as co-PI of PICANet. Hannah Mitchell is funded by an NIHR Academic Clinical Fellowship. The authors have no conflicts of interest relevant to this article to disclose.
