# Supplementary material for: Contribution of ethnicity, area level deprivation and air pollution to paediatric intensive care unit admissions in the United Kingdom 2008–2021
Source: eClinicalMedicine. 2024 Aug 16;75:102776. doi: 10.1016/j.eclinm.2024.102776 (PMC11377131; doi:10.1016/j.eclinm.2024.102776)
Supplement: Supplementary material [file mmc1.docx]

**Supplemental table 1: Crude incidence of admission to PICU by smaller ethnic groups (n=200,685 admissions)**

|  | Incidence | 95% CI |
| --- | --- | --- |
| White British | 120.0 | (119.3-120.6) |
| White Irish | 56.1 | (54.2-58.1) |
| Other White | 122.2 | (119.8-124.6) |
| Mixed | 74.0 | (72.3-75.7) |
| Asian Indian | 112.4 | (109.4-115.4) |
| Asian Pakistani | 189.2 | (185.9-192.6) |
| Asian Bangladeshi | 121.1 | (116.9-125.5) |
| Other Asian | 161.3 | (156.8-166) |
| Black Carribean | 166.1 | (159.1-173.3) |
| Black African | 155.1 | (151.8-158.6) |
| Black Other | 149.4 | (143.6-155.3) |
| Chinese | 85.0 | (79.2-91.1) |
| Arab | 12.3 | (6.4-21.6) |

**Supplemental table 2: Incidence rate ratio (IRR) of admission to PICU by ethnicity, deprivation and pollution for children admitted to PICU requiring for unplanned admission with a primary respiratory diagnosis or requiring invasive ventilation**

|  |  |  |  |  |  |  |  |
| --- | --- | --- | --- | --- | --- | --- | --- |
|  |  | Unplanned respiratory admissions | | | Ventilated admissions | | |
|  |  | IRR | 95% CI | P-value | IRR | 95% CI | P-value |
| Ethnicity^a^ | White | 1 (ref) | | | 1 (ref) | | |
|  | Asian | 1.34 | (1.29-1.41) | <0.001 | 1.18 | (1.15-1.21) | <0.001 |
|  | Black | 1.67 | (1.57-1.77) | <0.001 | 1.28 | (1.23-1.33) | <0.001 |
|  | Mixed | 0.60 | (0.56-0.65) | <0.001 | 0.53 | (0.51-0.56) | <0.001 |
|  | Other | 1.23 | (1.13-1.33) | <0.001 | 1.31 | (1.25-1.37) | <0.001 |
| Area level deprivation fifth^b^ | 1 (least deprived) | 1 (ref) | | | 1 (ref) | | |
|  | 2 | 1.02 | (0.97-1.06) | 0.44 | 1.09 | (1.06-1.12) | <0.001 |
|  | 3 | 1.10 | (1.05-1.15) | <0.001 | 1.20 | (1.17-1.24) | <0.001 |
|  | 4 | 1.29 | (1.23-1.35) | <0.001 | 1.38 | (1.34-1.41) | <0.001 |
|  | 5 (most deprived) | 1.63 | (1.56-1.71) | <0.001 | 1.50 | (1.46-1.54) | <0.001 |
| Air pollution (per 1mg/m3 increase)^c^ | | 1.01 | (1.00-1.01) | 0.046 | 1.02 | (1.02-1.02) | <0.001 |

**a Adjusted for sex and age (2008-2021)**

**b Adjusted for sex, age and ethnicity (2008-2021)**

**c Adjusted for sex, age, ethnicity and deprivation (2010-2021)**

**Supplemental table 3: Incidence rate ratio (IRR) of admission to PICU in Asian and Black children (white as reference) stratified by quintile of deprivation**

|  |  |  |  |  |  |  |  |
| --- | --- | --- | --- | --- | --- | --- | --- |
|  |  | Asian | | | Black | | |
|  |  | IRR | 95% CI | P-value | IRR | 95% CI | P-value |
| Area level deprivation Fifth | 1 (least deprived) | 1.11 | (1.02-1.22) | 0.014 | 1.49 | (1.33-1.67) | <0.001 |
|  | 2 | 0.99 | (0.92-1.07) | 0.854 | 1.34 | (1.20-1.50) | <0.001 |
|  | 3 | 1.04 | (0.98-1.11) | 0.149 | 1.23 | (1.15-1.32) | <0.001 |
|  | 4 | 1.12 | (1.07-1.18) | <0.001 | 1.18 | (1.10-1.26) | <0.001 |
|  | 5 (most deprived) | 1.15 | (1.11-1.20) | <0.001 | 1.24 | (1.17-1.31) | <0.001 |

**^a^ Adjusted for age and sex**

**Supplemental figure 1: directed acyclic graph of variables**

*
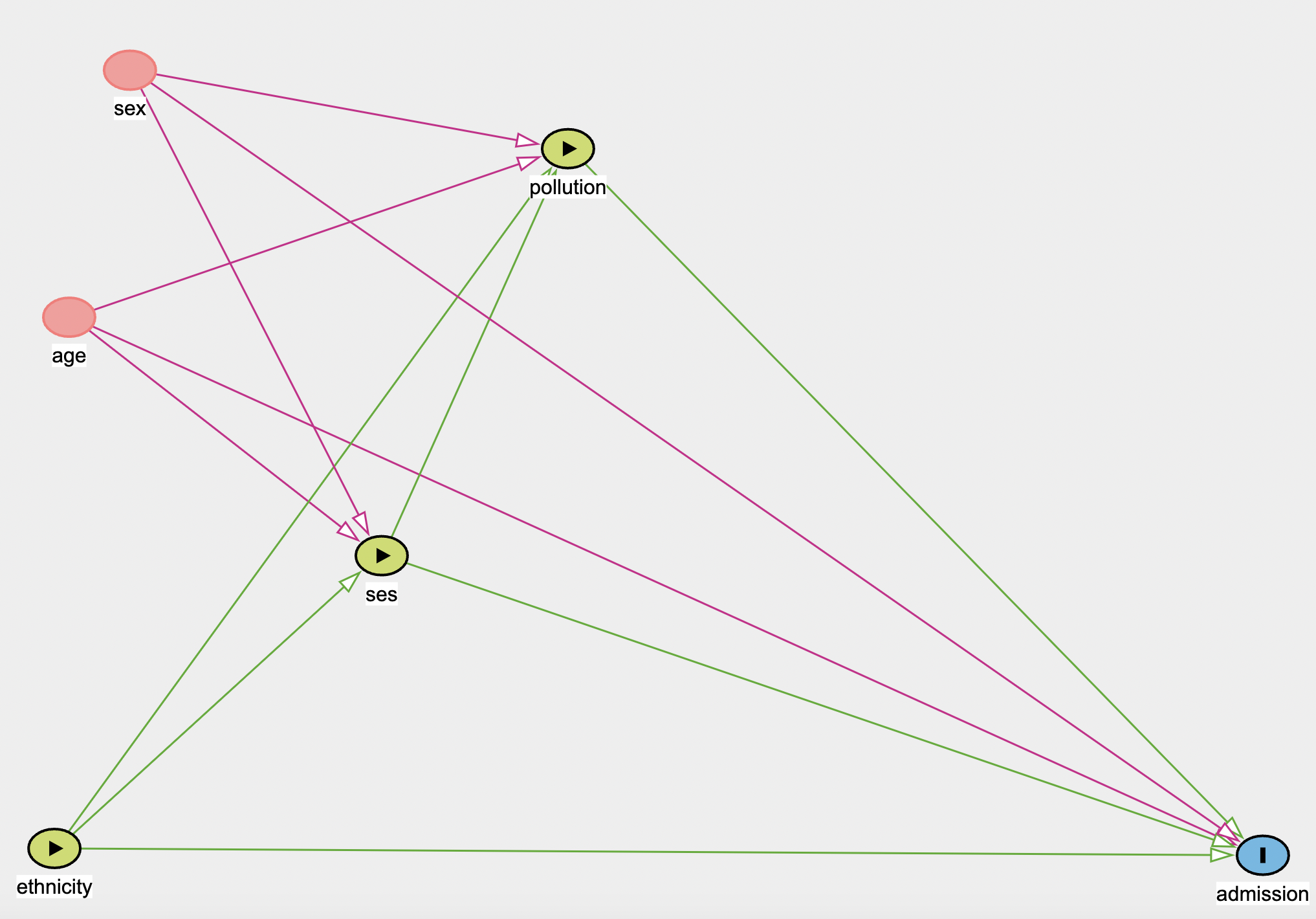
*
